# A synthesis and performance evaluation of a highly efficient ecological dust depressor based on the sodium lignosulfonate–acrylic acid graft copolymer

**DOI:** 10.1039/c7ra12556a

**Published:** 2018-03-22

**Authors:** Yanghao Liu, Wen Nie, Yubing Mu, Haihan Zhang, Hongkun Wang, Hu Jin, Zhiqiang Liu

**Affiliations:** College of Mining and Safety Engineering, Shandong University of Science and Technology Qingdao 266590 Shandong Province China niewen@sdust.edu.cn liuyanghao2012@163.com; State Key Laboratory of Mining Disaster Prevention and Control Co-found by Shandong Province and the Ministry of Science and Technology, Shandong University of Science and Technology Qingdao 266590 China

## Abstract

In this paper, a highly efficient and environmentally-friendly dust depressor was developed based on the sodium lignosulfonate–acrylic acid graft copolymer. Using the grafting ratio as an index, a three-factor and four-level orthogonal experiment was conducted to optimize the fabrication conditions of the graft copolymer. At a reaction temperature of 60 °C, feed ratio *m*_SLS–AA_ of 1 : 3, and FeSO_4_·7H_2_O content of 2.5%, the prototype produced the highest grafting ratio. The microstructure of the graft copolymer was measured using Fourier transform infrared spectrometry (FTIR) and nuclear magnetic resonance (^1^H-NMR). Furthermore, the influence of glycerol and the JFC penetrant on the contact angle between the compound solution and coal powder was investigated. Finally, four formulas of dust depressor were selected based on the experimental results. The dust-control performance of the four dust depressors was then tested on a large-scale spray dust suppression simulation platform. The results show that after applying formula 1 at various distances from the spray field, the average dust reduction rates of the total dust (respirable dust) at each point increased. Compared to the water-spraying dust suppression technique, the dust concentration is significantly reduced after the graft copolymer dust depressor is applied.

## Abstract 2

In this paper, a highly efficient and environmentally-friendly dust depressor was developed based on the sodium lignosulfonate–acrylic acid graft copolymer.

## Graphical abstract

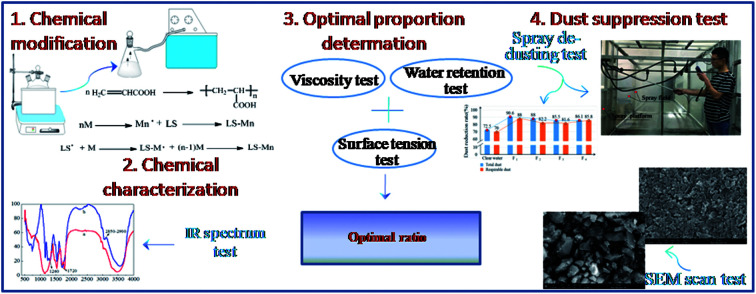

## Introduction

In recent years, there has been an ever-increasing environmental issue in industrial working areas, due to pollutants that consist mainly of inhalable particles (PM10) and fine particles (PM2.5). In the coal industry in particular, most of the respirable dust can enter directly into a human's lungs and form deposits there; furthermore they can gain access to the circulation system, causing severe hazards for the human body.^1–3^ Among the current dust suppression methods, spraying, using water as the liquid, has been applied extensively in underground coalmines. However, the coal dust's surface contains a large amount of the hydrophobic nonpolar group, leading to a strong hydrophobicity of the coal dust.^4^ Moreover, water's large surface tension leads to poor wettability with regard to the coal dust.^5–7^ Thus, most of the coal dust, particularly the respirable dust, does not become wet quickly and efficiently and is therefore not suppressed by water. Due to their small size, some of the dust particles deposited on the ground can also be dissipated easily by the wind, leading to secondary dust emissions. However, adding a dust depressor to water has proved to be an effective way of increasing the dust suppression rate by spraying. The development of a highly efficient dust depressor based on the dust's properties is therefore of great importance with regard to improving the dust control performance. To date, researchers worldwide have paid a great deal of attention to the development of a chemical dust depressor and there have been some notable achievements. For example, Kim J. *et al.*^8^ fabricated a dust depressor for wetting coal dust using a nonionic surfactant including surfynol 440, macol 30, plurafac RA 43, mindust 293, and neodol 92 as the main contents. Furthermore, Haining Wang *et al.*^9^ developed a dust binder from a starch grafted sodium polyacrylate. Cuifeng Du *et al.*^10^ synthesized a cohesive dust suppressant using a polymer film forming agent, polysaccharide polymer filler, and other auxiliaries. Miguel. A. Medeirosa *et al.*^11^ studied the dust suppressant fabricated from a glycerol polymerization and catalyzed by acids (sulfuric acid or phosphoric acid) and bases. Finally, Shuyan Cheng *et al.*^12^ created an environment-friendly dust depressor using byproducts from the production of biodiesel, such as glycerin.

**Fig. 1:**
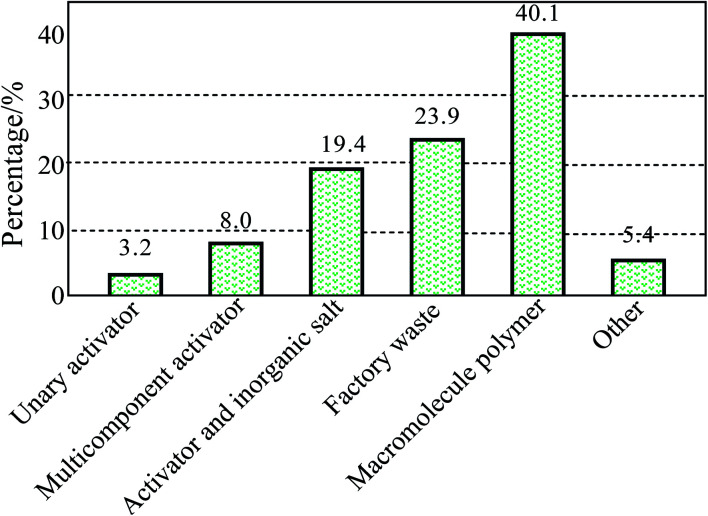
Statistics of various dust depressors from 2001 to 2009.

The main contents of the chemical dust depressors that emerged from the literature between 2001 and 2009 are shown in Fig. 1.^13,14^ As can be seen from this figure, the components of the chemical dust depressor have evolved from simple surfactants, along with their compounds and inorganic salts, to organic polymers. There is also an increasing tendency to use green, environmentally-friendly dust depressors. Therefore, combining the characteristics of organic polymers and environmentally-friendly dust depressors can promote their wider usage as a novel and highly efficient dust depressor.

As a byproduct from the papermaking industry, sodium lignosulfonate (LSL) benefits from its low cost, with an annual production of approximately 150 000 tons in China.^15,16^ Lignin is a renewable resource with good water solubility and cohesion, nontoxicity, and biodegradability. The active groups in SLS molecules such as hydroxyl and the sulfonic group can bond with coal particles and form a solidified layer on the coal's surface. After forming a sodium lignosulfonate–acrylic acid graft copolymer (GC_S–A_) with acrylic acid (AA), the surface tension is significantly reduced. In addition, AA has the structure of CH_2_=CH–COOH, where the carboxylic acid with a double bond is introduced to GC_S–A_. The carboxylic acid in a GC_S–A_ molecule provides a crosslinking point for the reaction. Moreover, the crosslinking structure formed from the reaction between carboxyl and hydroxyl in the molecule chain or SLS can greatly enhance the viscosity of the copolymer.^17–19^ Therefore, the inclusion of SLS in a chemical dust depressor has a number of advantages.

The graft copolymer of SLS and AA consists of two polymerization processes: the polymerization of AA grafting to SLS and the self-polymerization of AA.^20,21^ The self-polymerization of AA is a simple process, and the reaction equation is shown in eqn (1):1
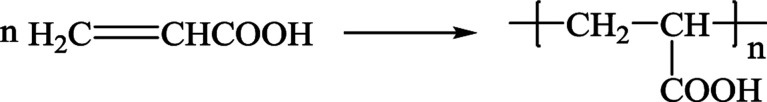


The graft polymerization of AA and SLS can take place at C-5 or in phenolic groups. As shown in eqn (2), SLS has a dynamic equilibrium. The graft polymerization of SLS and AA can occur in the following two pathways: (1) AA grafting to SLS following a one-by-one sequence, as shown in eqn (3); and (2) AA grafting to SLS after its self-polymerization, as shown in eqn (4).2
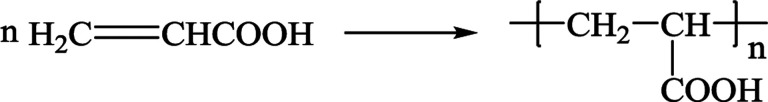

3 LS˙ + M → LS − M˙ + (*n* − 1)M → LS − Mn

4 *n*M → Mn˙ + LS → LS − Mn

Glycerin is a nontoxic, noncorrosive, and nonflammable liquid with a good moisture absorption capability, which satisfies the requirements of an ideal green solvent. The utilization of glycerin has recently become a focus of environmentally-focused chemistry. Previously, most of the studies in this area concentrated on the conversion of glycerin, while little has been explored with regard to utilizing it in new ways.^22^ It has been reported that glycerin can act as an auxiliary to increase the crosslinking degree among the large molecule chains of the graft copolymer, and expand the three-dimensional water-absorption space by forming a network structure.^23,24^ Therefore, in this paper, based on GS_S–A_, a new type of highly efficient and environmentally-friendly dust depressor is synthesized using glycerin as a wetting agent and the JFC penetrant as excipients.

## Experimental methods

### Raw materials and equipment

Sodium lignosulfonate (SLS, industrial grade), acrylic acid (AA, analytical purity), FeSO_4_·7H_2_O, acetone, glycerin, JFC penetrant, distilled water, magnetic stirrer with constant temperature and heater (85-1), water recycling vacuum pump (8HZ-D(III)), vacuum drying oven (DHG-9030), automatic tensionmeter (JK99C), nuclear magnetic resonance spectrometer (Bruker, AVANCE III 400, Swiss), FTIR spectrometer (Nicolet 6700), spin viscometer (NDJ-79).

### A synthesis of the graft copolymer

**Fig. 2:**
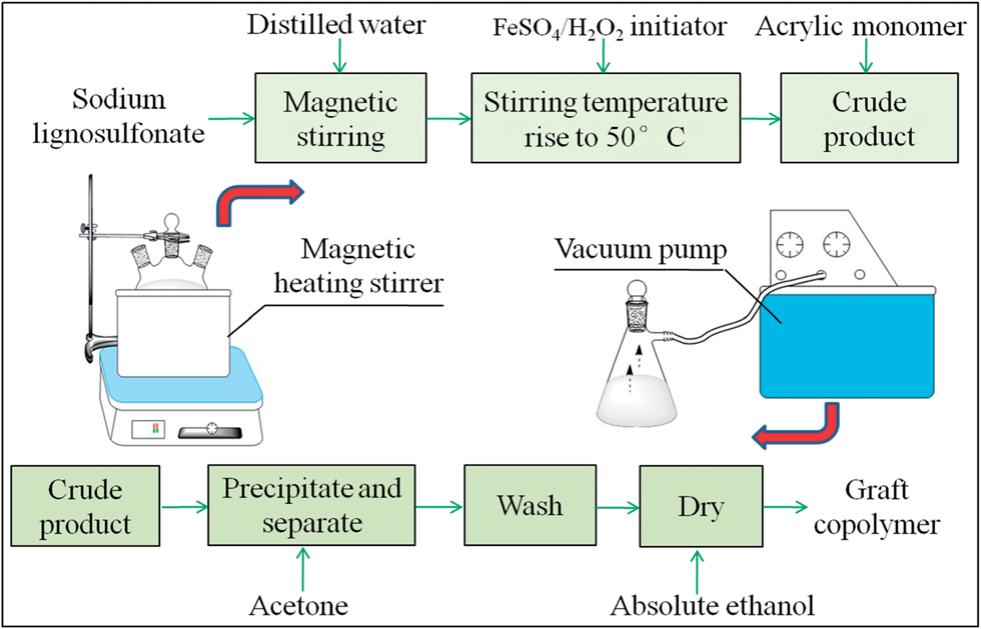
The reaction process of the SLS–AA graft copolymer.

The SLS solution and distilled water of a certain ratio were added to a three-neck flask (250 mL). After stirring for 10 min with a magnetic stirrer to activate the reactant, an initiator and AA were added to the solution. The reaction temperature was maintained at 50 to 80 °C, and the raw copolymer was obtained after 3 hours. The reaction process of the copolymer is shown in Fig. 2. After separation and purification, the product was weighed, and the GC_S–A_ yield was calculated following the process in ref. 25. The calculation equation is as follows:^26^5
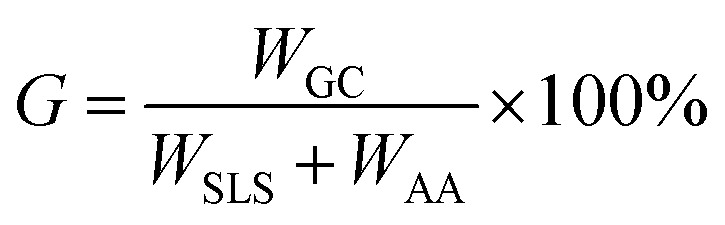
where *G* is the yield (%), *W*_GC_ is the mass (g) of GC_S–A_, *W*_SLS_ is the mass (g) of SLS, and *W*_AA_ is the mass (g) of glycerin.

### The design of the individual experiment

#### The surface tension of the SLS solution

An automatic tensionmeter (JK99C) was employed to measure the surface tension of the SLS solution. The tensionmeter has a measurement range of (0–1.8) × 10^3^ N cm^−1^ and an accuracy of 10^−6^ N cm^−1^. The surface tension of the SLS and GC_S–A_ solution with various mass concentrations was then measured. The optimized concentration range was determined after comparing the surface tension before and after the modification of the SLS. Each solution was measured three times, and the average value was used to ensure the accuracy of the results.

#### The viscosity of the GC_S–A_ solution

The cohesive force among the dust particles relies on the viscosity of the dust depressor. Thus, the viscosity is an important parameter for determining the dust depressor's practical application.^27^ In this paper, a spin viscometer (NDJ-79) was used to measure the viscosity of the GC_S–A_ solution with various mass concentrations. The #1 spinner was adopted with a rotation speed of 60 rpm. Each experiment was repeated three times to obtain the average value.

#### The water retention of coal dust with glycerin

As an auxiliary, glycerin can enhance the crosslinking degree among the large molecules of the graft copolymer, and increase the three-dimensional water-absorption space by forming a network structure. However, if overdosed, the stretching of the molecule chain can be limited, making it difficult to expand. In order to obtain the best concentration range, a water retention experiment was designed to test the water-holding capacity of coal powders, with various coalification degrees, under the presence of glycerin at various concentrations.

Six types of coal sample were selected based on the coalification degree: lignite coal (HM, Shenum, Shanxi, China), long flame coal (CY, Hebi, Henan, China), 1/3 coking coal (1/3JM, Huipodi, Shanxi, China), fat coal (FM, Jiangzhuang, Shandong, China), lean coal (PSM, Shiquan, Shanxi, China), and anthracite coal (WY, Tongzi, Guizhou, China). Each coal sample was collected according to the published national standard (GB/T482-2008) to ensure its representativeness.^28^ After being grinded to a coal powder by a vibration-grinding machine, the samples were put through a 200 mesh standard sieve, labeled, and stored at a low temperature.

The water retention test evaluates mainly the water-holding capacity of the solution, based on the variation trend of the water loss rate with time. The water loss rate can be calculated as follows:6
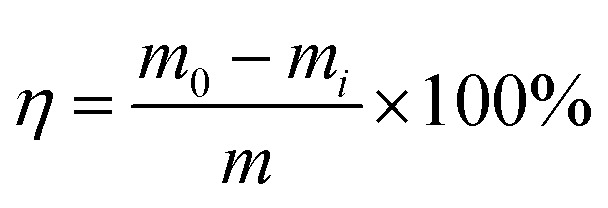
where *η* is the water loss rate (%), *m*_0_ is the initial mass (g) of the Petri dish, *m*_*i*_ is the mass (g) of the Petri dish after *i* hours, and *m* is the mass (g) of the 10 mL solution.

20 mg of each coal sample was placed in a 60 mm Petri dish, and 10 mL of glycerin at different mass concentrations was sprayed uniformly onto the dust sample. As a control experiment, water was also sprayed onto separate samples. Next, the samples were placed in a drying oven at 70 °C, with the Petri dish's mass measured hourly for 7 hours. The water loss rate was then calculated.

#### The individual permeability measurement

After pre-treatment (a 200 mesh sieve, dried at 105 °C, cool down), two parts of 15 g of coal dust were placed in individual 10 mm × 10 mm test tubes. The test tubes were vibrated until the dust filled 7.5 cm of the tubes. 24 mL of the JFC penetrant and distilled water were dropped slowly into the test tubes with burettes. The penetration depth of the solution as a function of time was recorded.

#### The orthogonal experiment

The concentration ranges obtained from each individual experiment were used to design an orthogonal experiment for selecting the optimized formula of the dust depressor. A three-factor and four-level orthogonal experiment was performed using a GC_S–A_ solution, glycerin, and the JFC penetrant as the three factors (noted as A, B, and C). A max–min difference analysis was conducted on the experimental value of the contact angle from each sample.

An optical droplet morphology analyzer (DSA100) was adopted to measure the dynamic contact angle between different solutions and the fat coal from Jiangzhuang. The contact angle between the coal and solution at 30 s was measured, and the experiment was repeated four times to obtain the average value for each solution.

### The chemical structure of the dust depressor

#### The nuclear magnetic resonance measurement

A nuclear magnetic resonance spectrometer (Bruker, AVANCE III 400 MHz, Swiss) was employed, which used TMS as an internal marker and deuterated water as a solvent. The hydrogen nuclear magnetic resonance spectra of the SLS and GC_S–A_ samples were measured.

#### The FTIR measurement

An FTIR spectrometer (Nicolet 6700) was used in this experiment. After dried in vacuum, the product was mixed with KBr at a ratio of 1 : 150. The FTIR spectrum of the mixed powder was then measured in the range of 4000 to 400 cm^−1^ with five scan times.^29,30^

#### The spray dust suppression simulation test

**Fig. 3:**
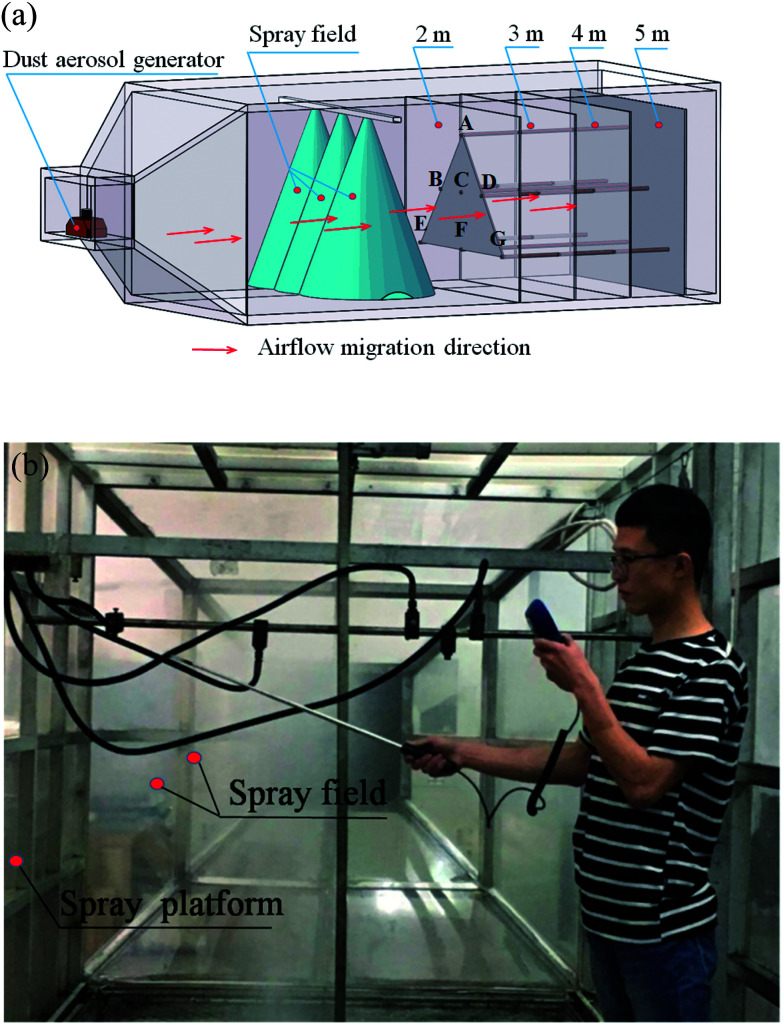
The spray dust suppression experiment: (a) the distribution of the measurement points and cross sections in the large-scale spray test box; (b) the field measurement of spray dust concentration.

The spray dust suppression simulation test was conducted on a large-scale mine spray simulation platform in the key state laboratories at the School of Mines, Shandong University of Science and Technology. The spray simulation setup consists of a rectangular test box of 6 m × 3 m × 2.5 m, a spray booster pump, water tanks, spray pipes, and high-pressure gauges. The spray nozzle was a single water swirl nozzle often used in coalmines. The atomizing form was an X shape diversion core flaring fitting with diameter of 2.4 mm. The spray pressure was set to 8 MPa, according to the general spray pressure used in China's coal mining equipment. In the simulation, the dust was emitted by a coal dust aerosol generator. To monitor the dust mass concentration accurately, cross sections at 2, 3, 4, and 5 m from the spray field were selected with 7 measurement points in each cross section, labeled A to G. The dust concentration was measured by a dust sampler for mines (AKFC-92A). Fig. 3(a) shows the three-dimensional distribution of the measurement points and cross sections in the test box. Fig. 3(b) shows a photo of the field dust concentration measurement.

#### Scanning electron microscope (SEM) experiment

The present experiment employs Nova Nano SEM 450 High-Resolution SEM to conduct observation over the surface morphology of two types of coal powder, *i.e.*, the dry coal powder without receiving dust suppressant treatment, and the coal powder with hardened film formed on the surface with the present hybrid dust suppressant applied. Before the experiment starts, the dry coal powder is sprayed with the dust suppressant, and placed in a drying chamber for desiccation such that a hardened film is formed on the sample surface. The resulting sample is then fixed to a sample holder with double-sided tape, which is in turn placed in a SEM sample chamber. After the vacuum is applied to the chamber, SEM images are taken.

#### The influence of dust suppression on the quality of coal

**Fig. 4:**
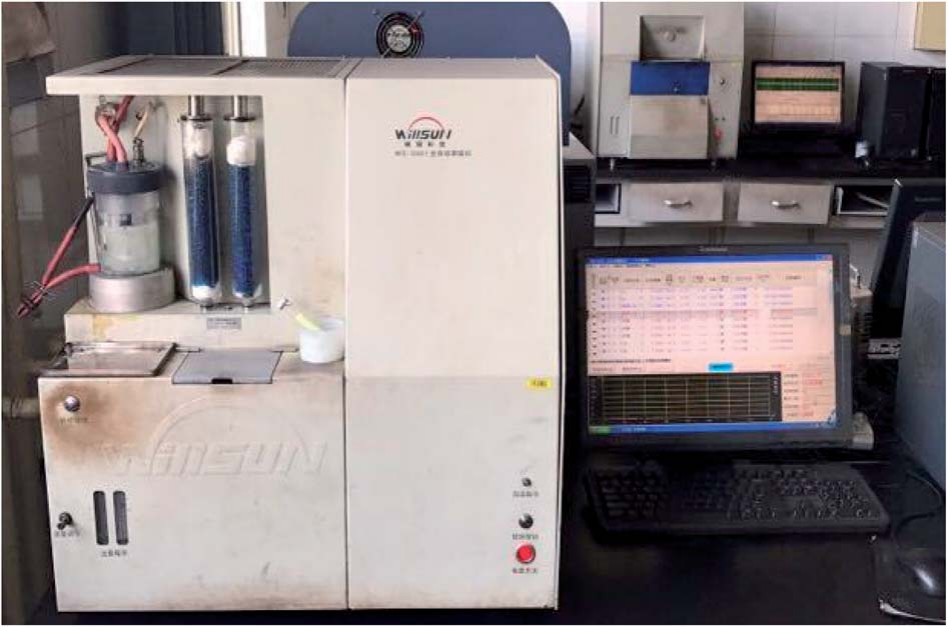
The automatic sulfur meter (WS-S501).

According to the coalification degree of the samples, four typical coal samples were selected. An automatic industrial analyzer (WS-G818), automatic sulfur meter (WS-S501), and automatic calorimeter (WS-C800) were used to measure the water content, dust content, volatile content, sulfur content, and calorific value of the coal powders before and after spraying the new compound dust depressor. In addition, the effect of the dust depressor on the quality of the coal was analyzed. Fig. 4 shows a photo of the automatic sulfur meter (WS-S501).

## Results and discussion

### The optimization of the graft copolymerization

In order to determine the optimum reaction conditions, based on the effect of various factors, the mass fraction and reaction time in this experiment were set as constant, and a three-factor and four-level orthogonal experiment was conducted to obtain the optimal experimental conditions. Here *A* denotes the reaction temperature (°C), *B* denotes the mass fraction of acrylic acid and sodium lignosulfonate, and *C* denotes the mass fraction (%) of FeSO_4_·7H_2_O. The experimental variable is the yield of the graft copolymer *G*, and the optimized condition has the largest *G*. The levels of the various factors in the orthogonal experiment are shown in Table 1, and the results are shown in Table 2.

**Table:** The levels of the various factors in the orthogonal experiment

Level	Factor		
	*A* (°C)	*B*	*C* (%)
1	50	1 : 2	2.5
2	60	1 : 3	3
3	70	1 : 3.5	3.5
4	80	1 : 4	4

The results of the orthogonal experiment

**Table:** 

No.	*A* (°C)	*B*	*C* (%)	*G* (%)
1	50	1 : 2	2.5	69.6
2	50	1 : 3	3	68.4
3	50	1 : 3.5	3.5	58.1
4	50	1 : 4	4	62.9
5	60	1 : 2	3	55.7
6	60	1 : 3	2.5	66.2
7	60	1 : 3.5	4	71.7
8	60	1 : 4	3.5	78.3
9	70	1 : 2	3.5	42.3
10	70	1 : 3	4	58.2
11	70	1 : 3.5	2.5	74.1
12	70	1 : 4	3	45.3
13	80	1 : 2	4	54.6
14	80	1 : 3	3.5	52.9
15	80	1 : 3.5	3	76.8
16	80	1 : 4	2.5	67.5

**Table:** 

K_1_	64.75	55.55	69.35	
K_2_	67.98	71.38	61.55	
K_3_	54.98	70.18	57.9	*A*_2_ *B*_2_ *C*_1_
K_4_	62.95	63.5	61.85	
R	13	15.83	11.45	

According to Table 2, the max–min difference analysis of the orthogonal experiment results shows that the main factor affecting the yield of the graft copolymer is the level of AA. A low AA level leads to its low concentration near the SLS molecules, and part of the SLS free radicals lose activity before the grafting reaction, resulting in a decreased yield. However, if the AA level is overly high, an AA self-polymerization reaction can occur, reducing the yield of the graft polymerization reaction between AA and SLS. As shown in Table 2, *A*_2_, *B*_2_, and *C*_1_ correspond to the highest graft yield. Thus, the optimized combination from the orthogonal experiment is *A*_2_*B*_2_*C*_1_. The effect of the three factors on the copolymer yield ranks as: *C* > *A* > *B*.

### The IR analysis of the graft copolymer

**Fig. 5:**
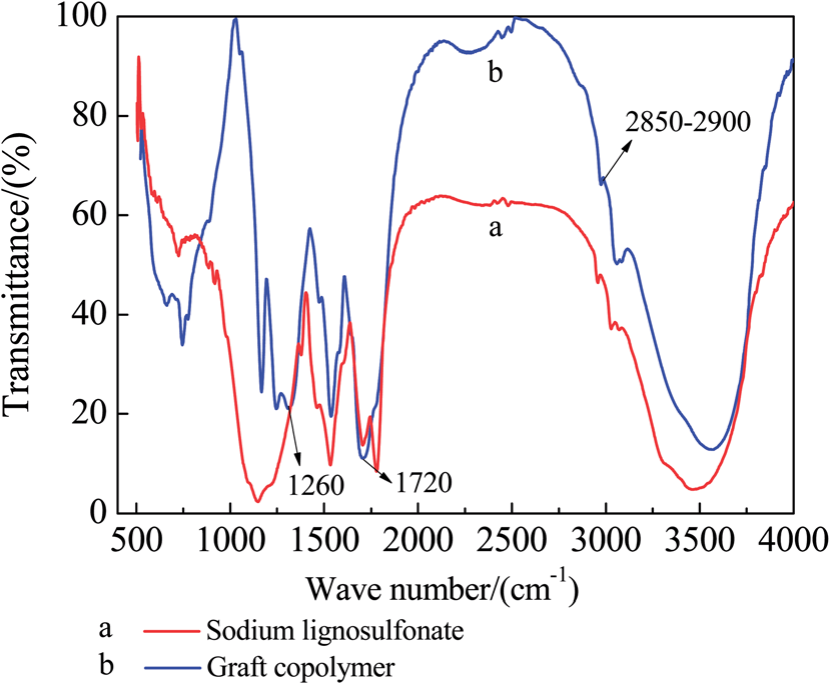
The FTIR of SLS and GC_S–A_.

Fig. 5 show the FTIR spectra of the SLS before and after the graft reaction. The comparison between the two figures shows that the grafted GC_S–A_ exhibits apparent hydroxyl absorption peak at 1720 cm^−1^, and the C–O stretching vibration peak at 1260 cm^−1^ is significantly enhanced, due to the carboxyl introduced to SLS after grafting AA. All the other features of the two spectra show no apparent difference, indicating that AA is grafted to SLS and forms graft copolymer.

### An analysis of ^1^H-NMR spectrum

**Fig. 6:**
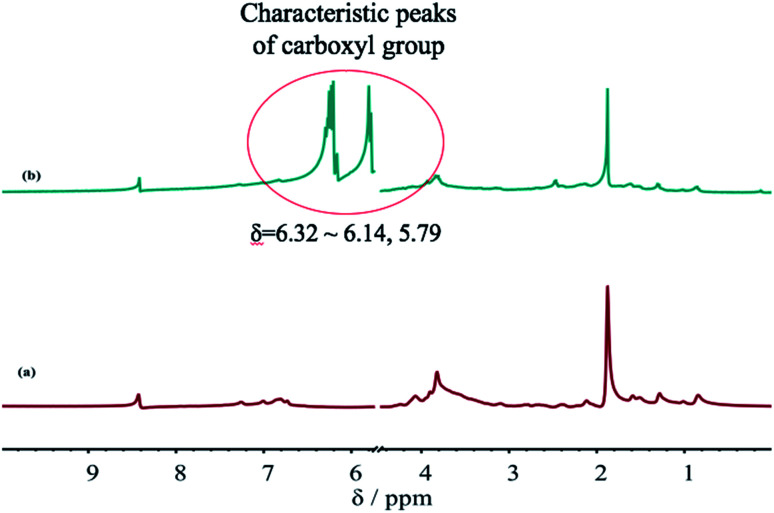
The ^1^H-NMR spectra of the SLS before and after grafting with the AA: (a) SLS; (b) GC_S–A_.

Fig. 6 shows the ^1^H-NMR spectra of the SLS before and after grafting with AA. A comparison between Fig. 6(a) and (b) shows a carboxyl (carbonyl) characteristic peak near *δ* = 6.32–6.14, 5.79 after the grafting reaction of SLS and AA, indicating that GC_S–A_ is successfully synthesized. Moreover, the results are consistent with the FTIR analysis. Meanwhile, the integral areas of hydrogen-related peaks in the ^1^H-NMR spectra suggest that the carboxyl (carbonyl) amount in the graft copolymer is significantly higher after the grafting reaction. This is because the double bond carboxylic acid in AA is introduced to the copolymer as a monomer, providing carboxyl for the copolymer molecule and crosslink points for the reaction. The carboxyl can react with the hydroxyl in the molecule chain or SLS, increasing the viscosity of the copolymer. Thus, GC_S–A_ can bond easily to a coal particle and enhance the capture rate of coal dust particles by the dust depressor.

### The surface tension and viscosity analysis

**Fig. 7:**
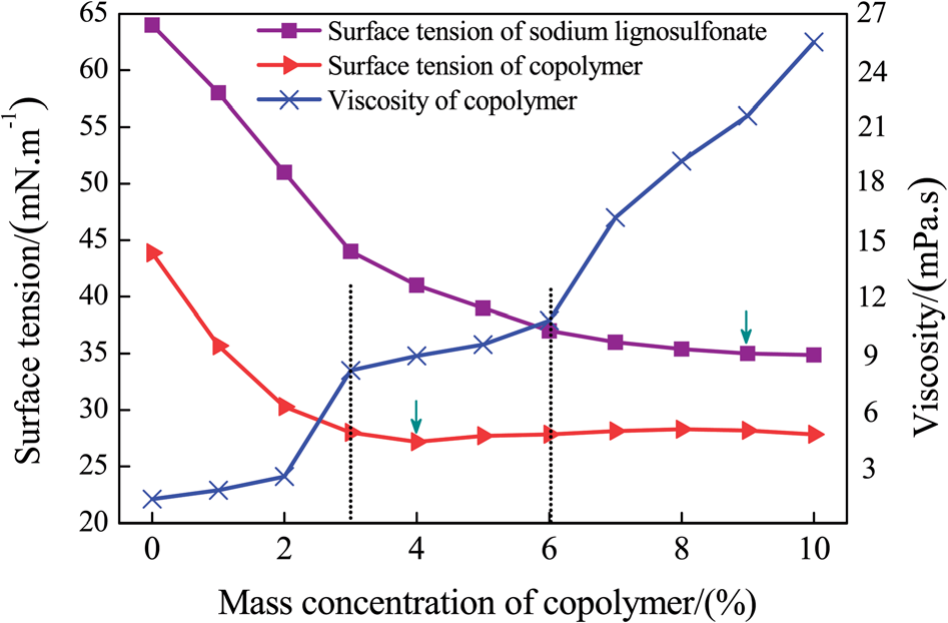
The viscosity and surface tension of the copolymer solution as a function of the mass fraction.

The wetting of the dust particles can be enhanced by modifying the surface tension between the liquid and solid–liquid. Meanwhile, the appropriate viscosity of the solution can increase its cohesive force on the coal powders. Thus, both the surface tension and viscosity are of great importance. In this paper, GC_S–A_ solutions with various mass fractions were prepared, and the viscosity and surface tension were measured under the same conditions. Having analyzed the experimental results, the viscosity and surface tension of the copolymer solution as a function of the mass fraction are shown in Fig. 7.

According to Fig. 7, at a room temperature of 25 °C and with an increase of the solution mass fraction, the surface tension of SLS and GC_S–A_ demonstrate a sharp initial decrease, followed by a mild decrease until a constant value is achieved. Before the grafting reaction, the surface tension of the SLS reaches a minimum value of 38.5 ± 0.5 mN m^−1^ with a mass fraction of 8–9%. After the grafting reaction, the surface tension of the GC_S–A_ reaches a minimum value of 26.7 ± 0.5 mN m^−1^ with a mass fraction of 3–4%. Thus, the surface tension of the SLS solution is greatly reduced after grafting with AA to form GC_S–A_. Meanwhile, the variation trend of the viscosity as a function of various mass fractions indicates that the viscosity increases significantly with the increase of the mass fraction. Nevertheless, a high viscosity may cause difficulties in the spraying process. Thus, further considerations should be taken into account to determine the best mass concentration range. The variation trend of the surface tension and viscosity of the GC_S–A_ solution suggest that both the surface tension and viscosity are within a relatively good region, as the mass fraction of the solution is 3–6%. Therefore, the preliminarily optimized mass fraction of the main material GC_S–A_ should be 3–6%.

### The influence of glycerin on the water retention of coal powders

**Fig. 8:**
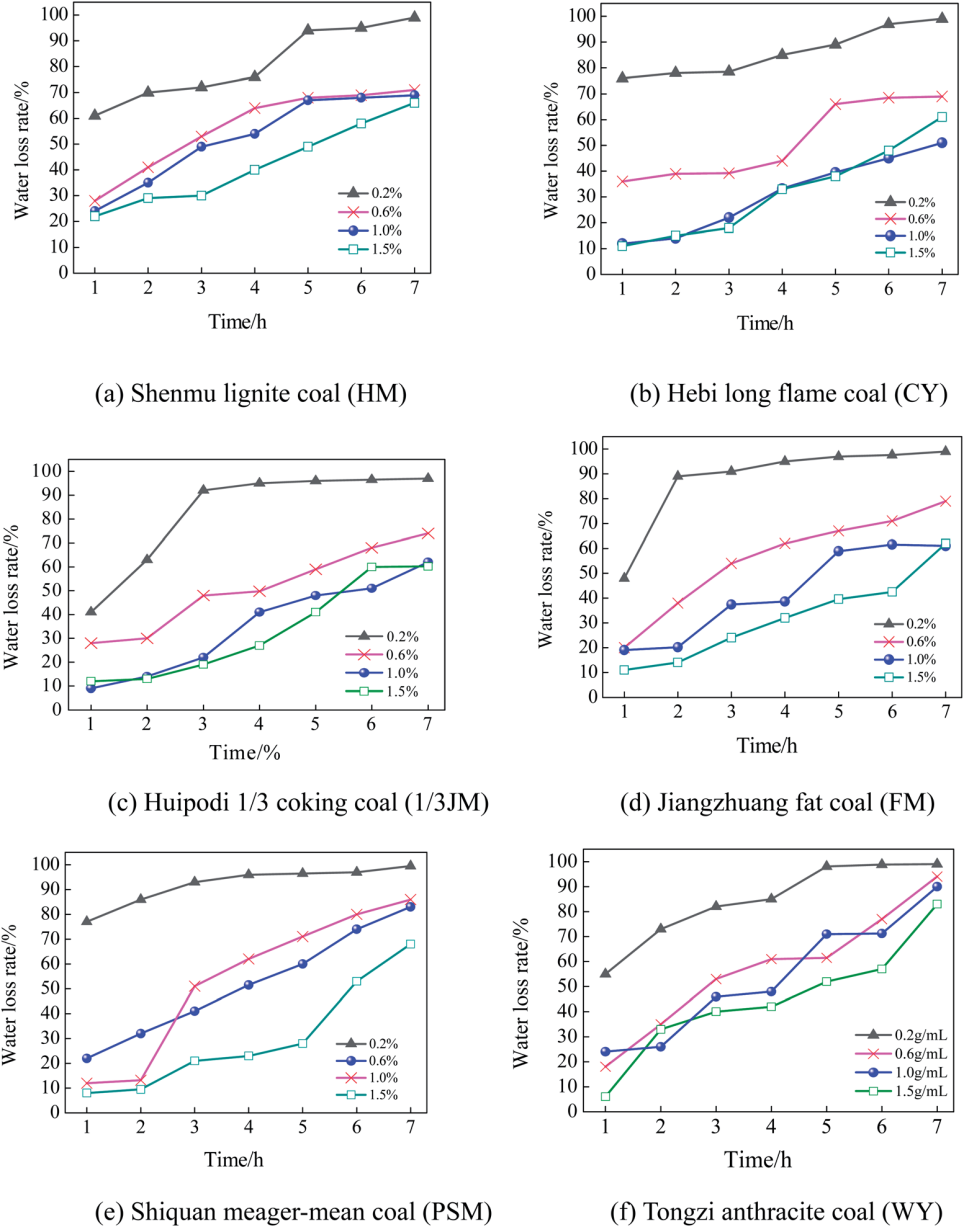
The influence of the glycerin concentration on the water retention performance of coal powders.

The glycerin additive can affect the water retention of coal powders. In order to determine the proper mass fraction for glycerin, six coal samples were selected based on their coalification degrees, and the water retention performance was measured with a glycerin mass fraction of 0.2%, 0.6%, 1.0%, and 1.5%. The results are shown in Fig. 8(a)–(e).

As shown in Fig. 8(a)–(e), glycerin has an apparent effect on the water retention performance of the coal powders. As the mass fraction of glycerin increases, the water retention is significantly enhanced. Nevertheless, as the mass fraction increases from 1.0% to 1.5%, the water retention ceases to further improve. As an auxiliary for a dust depressor, glycerin has a good moisture absorbance and water retention. However, an overdose of glycerin can limit the stretching of the molecule chain, reducing the ability to store water. In addition, as the coalification degree increases, the effect of glycerin on the water retention performance is reduced. As shown in Fig. 8(a) (HM sample) and 8(b) (CY sample), as the mass fraction of the spraying glycerin reaches 0.6–1.5%, the maximum water loss rate is below 70%. In Fig. 8(f) (WY sample), the water loss rate at 7 hours is above 90%. This is because as the coalification degree increases, the wetting becomes poor, leading to decreased water retention.

Overall, taking into account both water retention performance and ecological factors, the preliminary mass fraction of glycerin should be between 0.6% and 1.0%.

### An analysis of the penetration performance

**Fig. 9:**
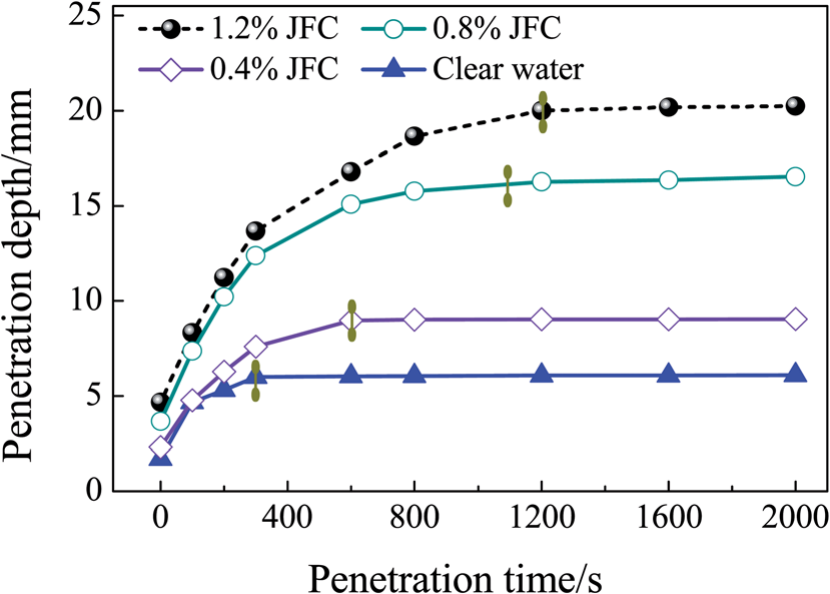
The penetration depth as a function of time.

Fig. 9 reflects the penetration depth as a function of time in the penetration test. According to Fig. 9, compared to the JFC penetrant, the penetration depth of pure water is significantly smaller. After a penetration time of 1200, 1100, and 600 s, JFC solutions with a mass fraction of 0.4%, 0.8% and 1.0% become stable. The penetration depth of pure water becomes stable at 300 s. Finally at 2000 s, the penetration depth of JFC solutions with a mass fraction of 0.4%, 0.8% and 1.0% is 3.32, 2.24, and 1.22 times the depth for pure water. Thus, the JFC possesses an apparent enhanced penetration performance. Moreover, the JFC solution with a mass fraction of 1.8% shows little difference when compared to the 1.0% solution. Therefore, taking into account ecological factors, the preliminary mass fraction of the penetrant should be 0.4% to 0.8%.

### An analysis of the orthogonal experimental results

In order to select the optimum formula for a dust depressor, an orthogonal experiment was designed, as shown in Table 3. A max–min difference analysis of the contact angle was conducted, and the results are shown in Table 4.

**Table:** The levels of the various factors in the orthogonal experiment

Factor	*A*	*B*	*C*
1	3%	0.6%	0.4%
2	4%	0.8%	0.5%
3	5%	0.9%	0.6%
4	6%	1.0%	0.8%

The results of the orthogonal experiment

**Table:** 

No.	*A*%	*B*%	*C*%	Contact angle/°
1	3	0.6	0.4	33.1
2	3	0.8	0.5	28.2
3	3	0.9	0.6	26.1
4	3	1	0.8	23.8
5	4	0.6	0.5	28.7
6	4	0.8	0.4	26.4
7	4	0.9	0.8	24.2
8	4	1	0.6	20
9	5	0.6	0.6	26.8
10	5	0.8	0.8	24.7
11	5	0.9	0.4	22.6
12	5	1	0.5	33.5
13	6	0.6	0.8	24.5
14	6	0.8	0.6	22.9
15	6	0.9	0.5	21.5
16	6	1	0.4	28.2

**Table:** 

K_1_	27.8	28.28	27.58	
K_2_	24.83	25.55	27.98	
K_3_	26.9	23.6	23.95	*BCA*
K_4_	24.28	26.38	24.3	
R	3.52	4.68	4.03	

K_1_, K_2_, K_3_, and K_4_ reflect the influence of various factors on the experimental index. A smaller contact angle between the dust depressor and coal powder is generally preferred. According to the results in Tables 3 and 4, *A*_4_, *A*_2_, *B*_3_, *C*_3_, and *C*_4_ are the optimum levels for each factor. A comparison of *R* values gives the following: *R*_B_ > *R*_C_ > *R*_A_. Thus, the influence of the factors on the contact angle ranks as *B* > *C* > *A*.

Overall, the optimum combinations of the three factors are *A*_4_*B*_3_*C*_3_, *A*_4_*B*_3_*C*_4_, *A*_2_*B*_3_*C*_3_, and *A*_2_*B*_3_*C*_4_. The four formulas of dust depressor determined by the orthogonal experiment are shown in Table 5.

**Table:** The formulas of the four dust depressors preliminarily selected by the orthogonal experiment

Formula	Component
1	6% *A* + 0.9% *B* + 0.6% *C*
2	6% *A* + 0.9% *B* + 0.8% *C*
3	4% *A* + 0.9% *B* + 0.6% *C*
4	4% *A* + 0.9% *B* + 0.8% *C*

### An analysis of the dust reduction rate

**Fig. 10:**
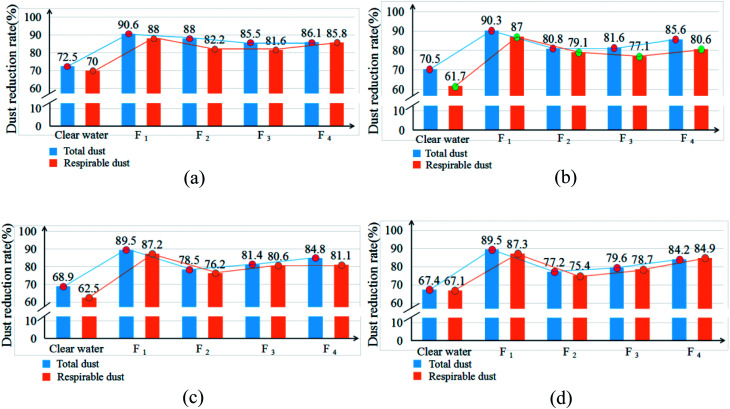
The average dust reduction rate of the total dust and respirable dust at the cross sections at different distances from the spray field.

The dust control performance of the four dust depressors selected by the orthogonal experiment were tested in a large-scale mine spraying test box in the key state laboratories at Shandong University of Science and Technology. At a spraying pressure of 8 MPa, the mass concentrations of the total dust and respirable dust were measured for each formula. Four different cross sections located at 2, 3, 4, and 5 m from the spray field were selected with 7 measurement points at each cross section. The average mass concentrations of the total dust and respirable dust were measured at each point. The results are shown in Table 6. Using the data in Table 6, the average dust reduction rates of the total dust and respirable dust for each formula were calculated, and the results are summarized in a line chart (Fig. 10).

**Table:** The average dust mass concentration at each point of the cross sections at various distances from the spray field

Distance to spray field/m	No dust reduction method		Clear water		Formula 1		Formula 2		Formula 3		Formula 4	
	*T*	*R*	*T*	*R*	*T*	*R*	*T*	*R*	*T*	*R*	*T*	*R*
2	812.6	426.1	266.7	127	76.4	51.1	97.5	75.8	117.8	78.4	113	60.5
3	729.5	364.2	215.2	139.5	70.5	47.2	140.1	76.1	134.2	83.4	106.5	70.7
4	642.4	291.6	199.8	109.4	67.5	37.3	138.1	69.4	122.5	56.6	97.6	55.1
5	541.6	233.1	176.6	76.7	59.4	29.5	123.5	57.3	110.5	49.7	85.6	35.2

According to Table 6 and Fig. 10, compared to water spraying dust suppression techniques, the dust reduction rates of the four chemical dust depressors in this paper are significantly higher. After using the spraying dust depressor of formula 1, the average mass concentrations of the total dust and respirable dust at 2, 3, 4, and 5 m from the spray field decrease from 812.6 and 426.1 to 76.4 and 51.1, respectively. The average dust reduction rates of the total dust and respirable dust at each point increase from 72.5% and 70% to 90.6% and 88%, respectively. Thus, the dust reduction performance of formula 1 is apparently higher than the other three formulas. Therefore, the highly efficient dust depressor in this paper reduces the dust mass concentration in the test box significantly, thereby improving the environmental quality.

### Analysis of SEM results

**Fig. 11:**
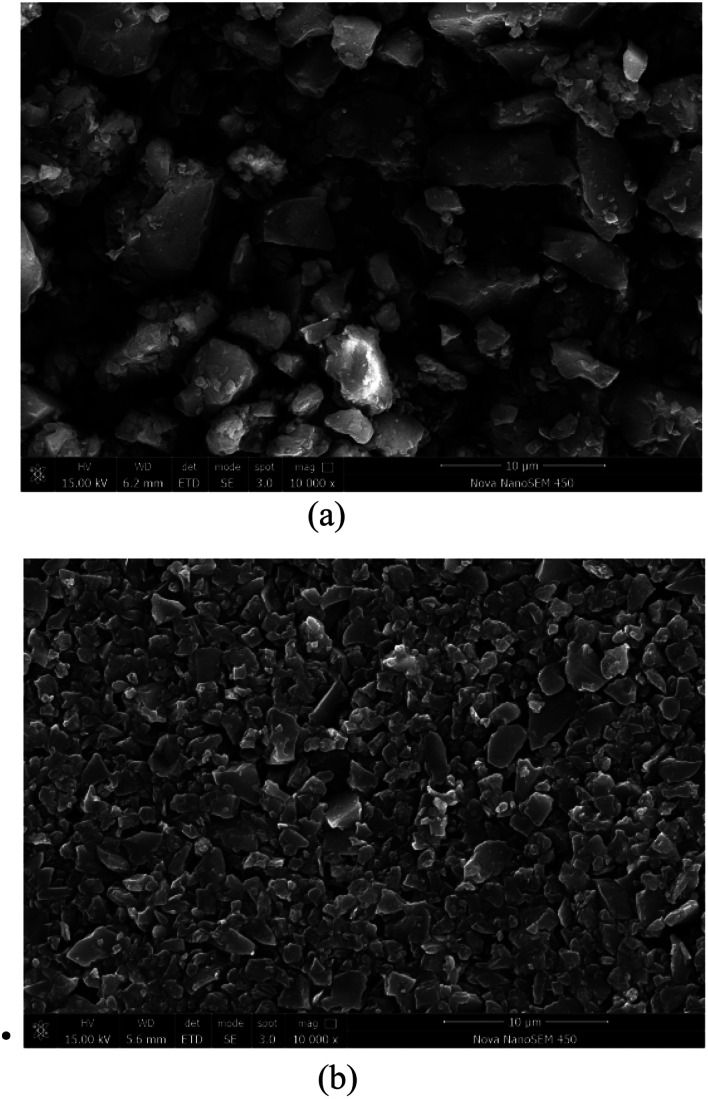
SEM photo of coal powder solidifying effect: (a) dry coal dust under 10 000× SEM; (b) coal dust after spraying dust suppressant under 10 000× SEM.

In order to investigate the effect of the new dust suppressant on the agglomeration and hardening of the coal powder, the present study uses high-resolution SEM to examine and compare the surface morphology of the dry coal powder without the spray of dust suppressant as well as the coal powder applied with the newly developed hybrid dust suppressant. The SEM images are shown in Fig. 11. Specifically, Fig. 11(a) depicts the surface morphology of the desiccated coal powder under 10 000× SEM without the application of dust suppressant. It can be found that the spacing between coal dust particles is relatively large, and no agglomeration pattern is observed. This is mainly due to the fact that the surface of the coal dust contains large amount of hydrophobic groups, and there lacks cementing effect between particles. Therefore, the distribution of particles is scattered, and consequently the coal dust has a strong tendency to undergo dispersion driven by wind, leading to severe pollution. Fig. 11(b) shows the 10 000× SEM image of the coal power undergoing the treatment of the new hybrid dust suppressant followed by desiccation. It is evident that the surface of coal powder is relatively smooth, and the coal dust particles are tightly connected with small spacing. This is due to the fact that the dust suppressant coats the coal dust surface with a hardened film. The film gives rise to a decent cementing effect between dust particles, rendering the coal dust particles a strong tendency to experience agglomeration. The reentrainment of coal dust is thereby suppressed.

### An analysis of coal quality

According to the general regulation of People's Republic of China national standard coal analysis and test method, the coal dust samples before and after spraying the dust suppressant were analyzed. Four representative coal samples from different regions were selected for determination depending on the degree of coalification. They are long flame coal (CY) from Shanxi Daliuta coal mine, 1/3 coking coal (1/3 JM) from Shanxi Huipodi coal mine, gas-fat coal (FM) from Shandong Jiangzhuang coal mine and anthracite coal (WY) from Guizhou Tongzi coal mine. Table 7 is the analysis results of coal dust before and after spray dust suppressant developed in this paper.

**Table:** Results of coal dust before and after spray dust suppressant developed in this paper^a^

Coal sample		*W*_B_%					Calorific value
		Total moisture	Analytical water	Ash content	Volatile matter	Total sulfur	
		*M*_t_	*M*_ad_	*A*_d_	*V*_d_	*S*_t,d_	*Q*_gr,d_ (MJ kg^−1^)
CY	1^#^	19.41	5.56	6.22	33.25	0.48	25.84
	2^#^	20.79	3.45	6.31	33.74	0.476	25.68
1/3JM	1^#^	16.5	1.85	2.8	60.2	0.52	34.01
	2^#^	19.21	1.77	2.77	59.68	0.49	32.99
FM	1^#^	17.22	1.55	5.6	30.56	0.39	35.62
	2^#^	19.56	1.53	5.64	30.47	0.38	34.87
WY	1^#^	14.32	2.69	7.21	9.64	0.35	40.01
	2^#^	17.3	2.7	7.18	9.39	0.33	39.55

a 1^#^ and 2^#^ respectively represent the samples of raw coal and the coal simple after spraying dust suppressant.

The comparison data in Table 7 indicate that after spraying the dust suppressant, the total moisture content of the coal sample increases, this is because the dust suppressant is an aqueous solution, which brings the total moisture content of the surface coal after spraying the dust suppressant; similarly, the coal total moisture also increased coal calorific value slightly causes. The reduced calorific value is all less than 0.1%, the effects are almost negligible. In addition, the whole water section of the coal surface will gradually evaporate during transportation, the resulting increase in moisture is also eliminated. Futhermore, spraying dust suppressant has no influence on ash content, volatile content and total sulfur content of coal. Therefore, the new hybrid dust suppressant developed in this paper will not affect the quality of coal.

## Conclusions

In this paper, a sodium lignosulfonate–acrylic acid graft copolymer was synthesized using low-cost polymer sodium lignosulfonate. The optimized reaction condition determined by the orthogonal experiment was as follows: a reaction temperature of 60 °C, feed ratio *m*_SLS–AA_ of 1 : 3, and FeSO_4_·7H_2_O content of 2.5%. The FTIR spectrum confirms the introduction of AA into SLS, the ^1^H-NMR spectrum shows that the variation of the group peak position for the graft copolymer is consistent with the FTIR results. Meanwhile, the carboxyl group on the copolymer has a crosslinking structure with the molecular chain or the hydroxyl group on SLS, which can increase the viscosity of the polymer. Four kinds of dust reducing agents were selected through water conservation experiment and permeability test, finally, the optimum formula was determined by the spray dust reduction experiment. From an analysis of the experimental results, the new dust depressor developed in this paper has no effect on the quality of the coal. In summary, the novel, ecological, highly efficient dust depressor developed in this paper is environmentally-friendly and easily degradable, with a significantly enhanced dust reduction performance.

## Conflicts of interest

There are no conflicts to declare.

## Supplementary Material
